# Efficacy and safety of TACE combined with lenvatinib and PD-1 Inhibitor in intermediate-stage HCC exceeding the up-7 criteria: a retrospective cohort study

**DOI:** 10.3389/fimmu.2025.1560750

**Published:** 2025-06-12

**Authors:** Miao Xue, Yanqin Wu, Yiyang Tang, Kun Huang, Haikuan Liu, Bowen Zhu, Jie Wen, Yue Zhao, Guixiong Zhang, Hang Liu, Wenzhe Fan, Jiaping Li

**Affiliations:** ^1^ The First Affiliated Hospital of Sun Yat-sen University, Guangzhou, China; ^2^ Guizhou Provincial People’s Hospital, Guiyang, China

**Keywords:** hepatocellular carcinoma, up-7 criteria, transarterial chemoembolization, lenvatinib, PD-1 inhibitor, combined therapy, tumor response

## Abstract

**Purpose:**

This study aimed to assess the efficacy and safety of combining transarterial chemoembolization (TACE) with lenvatinib and a PD-1 inhibitor (TACE+LEN+PD-1) compared to TACE combined with lenvatinib alone (TACE+LEN) in intermediate-stage hepatocellular carcinoma (HCC) patients exceeding the up-to-7 criteria

**Materials and methods:**

Data from 115 patients with intermediate-stage HCC exceeding the up-to-7 criteria, treated between January 2015 and December 2023, were prospectively collected and retrospectively analyzed. Key clinical outcomes, including overall survival (OS), progression-free survival (PFS), tumor response rates based on modified Response Evaluation Criteria in Solid Tumors (mRECIST), and adverse events (AEs), were evaluated and compared between the two treatment groups. Univariate and multivariate analyses were performed to identify factors affecting OS and PFS.

**Results:**

Among the patients, 35 received TACE+LEN+PD-1, and 80 underwent TACE+LEN. The TACE+LEN+PD-1 group achieved a longer median PFS (10.0 months vs. 5.7 months; *P*=0.002) and a median OS of 21.0 months, compared to 16.2 months in the TACE+LEN group, though the OS difference was not statistically significant (*P*=0.096). Progression to macrovascular invasion (MVI) or extrahepatic spread (EHS) was delayed in the TACE+LEN+PD-1 group compared to the TACE+LEN group (12.0 months vs. 7.5 months; *P*=0.007). Multivariate analysis identified treatment modality and tumor burden score (TBS) as independent prognostic factors for OS and PFS. Subgroup analyses showed that patients with an Eastern Cooperative Oncology Group (ECOG) performance status (PS) of 0 or HBV positivity derived greater benefits from TACE+LEN+PD-1, while those with high TBS or a Child-Pugh score of 7 did not show similar advantages. The rates and severity of AEs were comparable between groups (any grade: 88.6% vs. 91.3%, *P*=0.733; grade 3 or 4: 48.6% vs. 42.5%, *P*=0.546).

**Conclusion:**

TACE+LEN+PD-1 significantly improved PFS, particularly by delaying progression to MVI or EHS after the first TACE session in intermediate-stage HCC patients exceeding the up-to-7 criteria, compared to TACE+LEN. Subgroup analysis indicated superior survival benefits for patients with a PS of 0 or HBV positivity, but not in those with high TBS or a Child-Pugh score of 7. The safety profile of TACE+LEN+PD-1 was comparable to TACE+LEN. However, the OS benefit between the two groups was not statistically significant.

## Introduction

Hepatocellular carcinoma (HCC) ranks among the leading causes of cancer-related deaths globally, with over 50% of cases occurring in China. The majority of HCC patients in China are diagnosed at intermediate or advanced stages, primarily due to hepatitis B virus (HBV) infection, the dominant etiological factor (1). For intermediate-stage HCC, the China Liver Cancer Staging (CNLC) system currently recommends transarterial chemoembolization (TACE) as the standard treatment (2). However, its efficacy in patients with intermediate-stage HCC and high tumor burden has been increasingly questioned (3).

The up-to-7 criteria, originally designed as a prognostic tool to evaluate liver transplantation eligibility in HCC patients, have also become a common method for assessing tumor burden in intermediate-stage cases (4, 5). These criteria stipulate that the sum of the largest tumor diameter (in centimeters) and the number of tumors should not exceed seven. According to the Asia-Pacific Primary Liver Cancer Expert (APPLE) consensus, TACE alone is not recommended for treating intermediate-stage HCC patients with tumors that exceed the up-to-7 criteria (6). Consequently, TACE alone should not be used as first-line therapy in patients who do not meet these criteria. Furthermore, TACE combined with sorafenib represents a viable treatment option for patients with intermediate-stage HCC, particularly those exceeding the up-to-7 criteria (7). The final outcomes of the TACTICS trial suggested that combining TACE with systemic therapy may be necessary for patients whose tumors exceed the up-to-7 criteria.

Lenvatinib, a multikinase inhibitor, exerts its effects by targeting several critical receptors, including vascular endothelial growth factor (VEGF) receptors 1-3, fibroblast growth factor (FGF) receptors 1-4, platelet-derived growth factor (PDGF) receptor alpha, KIT, and rearranged during transfection (RET) (8). The REFLECT trial demonstrated that lenvatinib is non-inferior to sorafenib regarding overall survival (OS), leading to its recommendation as a first-line treatment for advanced HCC. Moreover, the LAUNCH trial underscored the potential benefits of combining TACE with lenvatinib as a first-line therapy for advanced-stage HCC (9). Additionally, findings from a phase 2, prospective, multicenter, single-arm trial provided encouraging evidence of the efficacy and safety of TACE combined with lenvatinib in unresectable intermediate-stage HCC patients who are not suitable candidates for TACE monotherapy (10). These results suggest that TACE combined with lenvatinib could serve as a viable treatment strategy for patients with intermediate-stage HCC exceeding the up-to-7 criteria.

The introduction of atezolizumab and bevacizumab as first-line treatments for unresectable HCC has paved the way for integrating immunotherapy into HCC treatment regimens (11). Additionally, a phase Ib study demonstrated that lenvatinib combined with pembrolizumab shows promising antitumor activity with manageable toxicities in patients with unresectable HCC (12). In a retrospective cohort study, TACE combined with lenvatinib and a programmed cell death 1 (PD-1) inhibitor significantly improved survival and tumor response compared to TACE combined with lenvatinib in advanced HCC patients, especially those with extrahepatic metastasis or multiple tumors (13). Despite these advances, the role of PD-1 inhibitors in intermediate-stage HCC, particularly in cases exceeding the up-to-7 criteria, remains controversial. This uncertainty arises from the lack of definitive evidence on treatment sequencing and head-to-head comparisons, making the choice between immunotherapy and kinase inhibitors largely empirical. Given the promising survival benefits observed with the combination of TACE and lenvatinib (TACE+LEN) in intermediate-stage HCC patients exceeding the up-to-7 criteria, there is ongoing debate over whether the addition of a PD-1 inhibitor to this regimen is justified.

We hypothesize that a comprehensive treatment approach combining TACE with lenvatinib and a PD-1 inhibitor (TACE+LEN+PD-1) could serve as a more effective therapeutic strategy for intermediate-stage HCC patients exceeding the up-to-7 criteria. This retrospective analysis aims to assess the efficacy and safety of TACE+LEN+PD-1 compared to TACE+LEN in this patient population.

## Materials and methods

### Study design and patient selection

This study was approved by the ethics committee of our hospital and conducted in accordance with the Declaration of Helsinki. The requirement for written informed consent was waived because this study was retrospective. The diagnosis of intermediate-stage HCC exceeding the up-to-7 criteria was confirmed according to the CNLC algorithm and the APPLE consensus. Patients were grouped based on the initial treatment modality selected at the time of diagnosis. From January 2015 to December 2023, a total of 193 patients with intermediate-stage HCC exceeding the up-to-7 criteria, who had selected either TACE+LEN+PD-1 or TACE+LEN as their initial therapy, were retrospectively reviewed at the first affiliated hospital of Sun Yat-sen university in China.

The eligibility criteria were: 1) a diagnosis of intermediate-stage HCC beyond the up-to-7 criteria confirmed by imaging and/or pathological diagnosis; 2) age 18–80 years with a life expectancy of at≥3 months (3); CNLC Stage Ib/IIa/IIb; 4) Eastern Cooperative Oncology Group (ECOG) performance status (PS) (14) of 0–1 before TACE, and Child–Pugh class (15) A or B7; 5) normal coagulation or renal function, corrected by appropriate treatment.

The exclusion criteria included: 1) a tumor burden exceeding 70% of the whole liver, diffuse type HCC; 2) having received previous systemic therapy, TACE, or radioactive seed implantation; 3) having received other anti-tumor treatments during the follow-up period, including local ablation, radioactive seed implantation, surgical resection, or other systemic therapy; 4) pulmonary fibrosis or autoimmune disease; 5) severe renal dysfunction, coagulation disorders, or cardiopulmonary dysfunction that cannot be corrected; 6) incomplete data.

### TACE procedure

TACE was administered based on patient preference for either conventional TACE (cTACE) or drug-eluting bead TACE (DEB-TACE). For cTACE, a mixture of lipiodol (Hengrui, Suzhou, China) and epirubicin (Pfizer, New York, USA) was infused into the tumor’s blood supply, followed by embolization using absorbable gelatin sponge (Alicon, Hangzhou, China). For DEB-TACE, drug-eluting beads like Callispheres (Hengrui, Suzhou, China) or DC Bead (Boston Scientific, Natick, USA) were employed, with epirubicin loaded into the beads before administration. In cases of residual tumors, additional embolization with absorbable gelatin sponge was performed. In situations where arterioportal or arteriovenous fistulas were present, the fistulas were first treated with embolization using 350-560 µm absorbable gelatin sponge before the introduction of the drug-oil emulsion or drug-loaded beads.

The multidisciplinary liver cancer team determined the overall treatment strategy, and experienced interventional radiologists (WZ Fan, JP Li) performed the TACE procedures. These procedures involved selective catheterization to pinpoint the feeding arteries, with superselective catheterization performed whenever possible. The choice between DEB-TACE and cTACE was guided by the operator’s expertise, and the size of the embolic materials ranged from 100µm to 500µm, chosen based on intraoperative findings. The embolization endpoint was marked by stasis in the tumor-feeding artery over 3 to 4 cardiac cycles, adhering to the Chinese clinical guidelines for TACE (16). TACE sessions were repeated when necessary, based on the presence of viable tumor tissue as determined by follow-up imaging such as computed tomography (CT) or magnetic resonance imaging (MRI), assuming the patient’s overall condition and organ function remained stable.

### Lenvatinib and PD-1 inhibitor administration

Lenvatinib (Eisai, Tokyo, Japan) and a PD-1 inhibitor were introduced within 7 days following the initial TACE procedure. Lenvatinib was administered orally at a dose of 12 mg daily for patients weighing 60kg or more, and 8 mg daily for those under 60kg. The PD-1 inhibitors–pembrolizumab (Merck & Co., New Jersey, USA) sintilimab (Innovent Biologics, Suzhou, China), tislelizumab (BeiGene, Shanghai, China), or camrelizumab (Hengrui, Suzhou, China) –were administered intravenously at a dose of 200 mg every three weeks. Adjustments or discontinuation of these medications were made based on the occurrence and severity of any drug-related adverse events (AEs), following the drug guidelines. This treatment was continued without interruption during the perioperative period of repeated TACE.

### Follow-up and tumor burden score

Baseline data, follow-up laboratory results, imaging results (such as contrast-enhanced abdominal CT, MRI, and chest CT), and clinical events (e.g., refractory ascites, gastrointestinal bleeding, and liver failure), were analyzed to assess the differences in efficacy and safety between the TACE+LEN+PD-1 or TACE+LEN group. The tumor burden score (TBS) is a prognostic marker for HCC undergoing TACE, calculated using the maximum tumor size and number of tumors. TBS was calculated using the following formula: TBS^2^= (maximum tumor diameter)^2^ + (number of tumors)^2^, as previously reported. A TBS greater than 13.74 is considered high, while a TBS less than 13.74 is considered low (17). Patients were followed up every 6 to 8 weeks, with blood tests conducted within 3 days before TACE and 2 days after TACE. The final follow-up was conducted on December 31 2023. During follow-up, TACE was repeated according to radiological assessment and patient condition. Besides, the treatment was discontinued in cases of intolerable toxicity, disease progression, or changes in the treatment plan.

Tumor response was assessed through CT or MRI imaging, using the modified Response Evaluation Criteria in Solid Tumors (mRECIST) (18). Liver function was evaluated based on the Child-Pugh classification (15). The decision for repeated TACE sessions was guided by a combination of tumor response, liver function status, and ECOG PS. Overall survival (OS) was defined as the interval between the first TACE session and either the date of death or the last follow-up. Progression-free survival (PFS) referred to the time from the initial TACE session to radiologic disease progression, as determined by mRECIST criteria, or death from any cause. PFS1 specifically represented the time from the first TACE session to intrahepatic tumor progression, whereas PFS2 marked the time from the initial TACE session until stage progression, which included the development of macrovascular invasion (MVI) or extrahepatic spread (EHS). The objective response rate (ORR) was defined as the proportion of patients achieving either complete response (CR) or partial response (PR), while the disease control rate (DCR) encompassed the sum of ORR and stable disease (SD), in accordance with mRECIST criteria (18).

### Statistical analyses

Categorical variables were expressed as patient numbers and corresponding percentages, while continuous variables were represented as mean ± standard deviation for data with normal distribution and as median (range) for data without normal distribution. Comparisons of categorical variables were conducted using the chi-square test or Fisher’s exact test, depending on the context. For continuous variables, Student’s t-test was applied to normally distributed data, whereas the Mann-Whitney U test was used for non-normally distributed data. The Kaplan-Meier method was employed to estimate survival curves, with group differences evaluated using the log-rank test. Variables showing a p-value<0.10 in univariate analysis were included in a multivariate Cox proportional hazards regression model to determine independent prognostic factors for OS and PFS. Statistical analyses were performed using SPSS Statistics (version 22.0, IBM, Armonk, New York, USA) and GraphPad Prism (version 10.0, GraphPad Software, San Diego, California, USA). All tests were two-sided, with statistical significance defined as a p-value<0.05.

## Results

### Baseline characteristics

**Figure 1:**
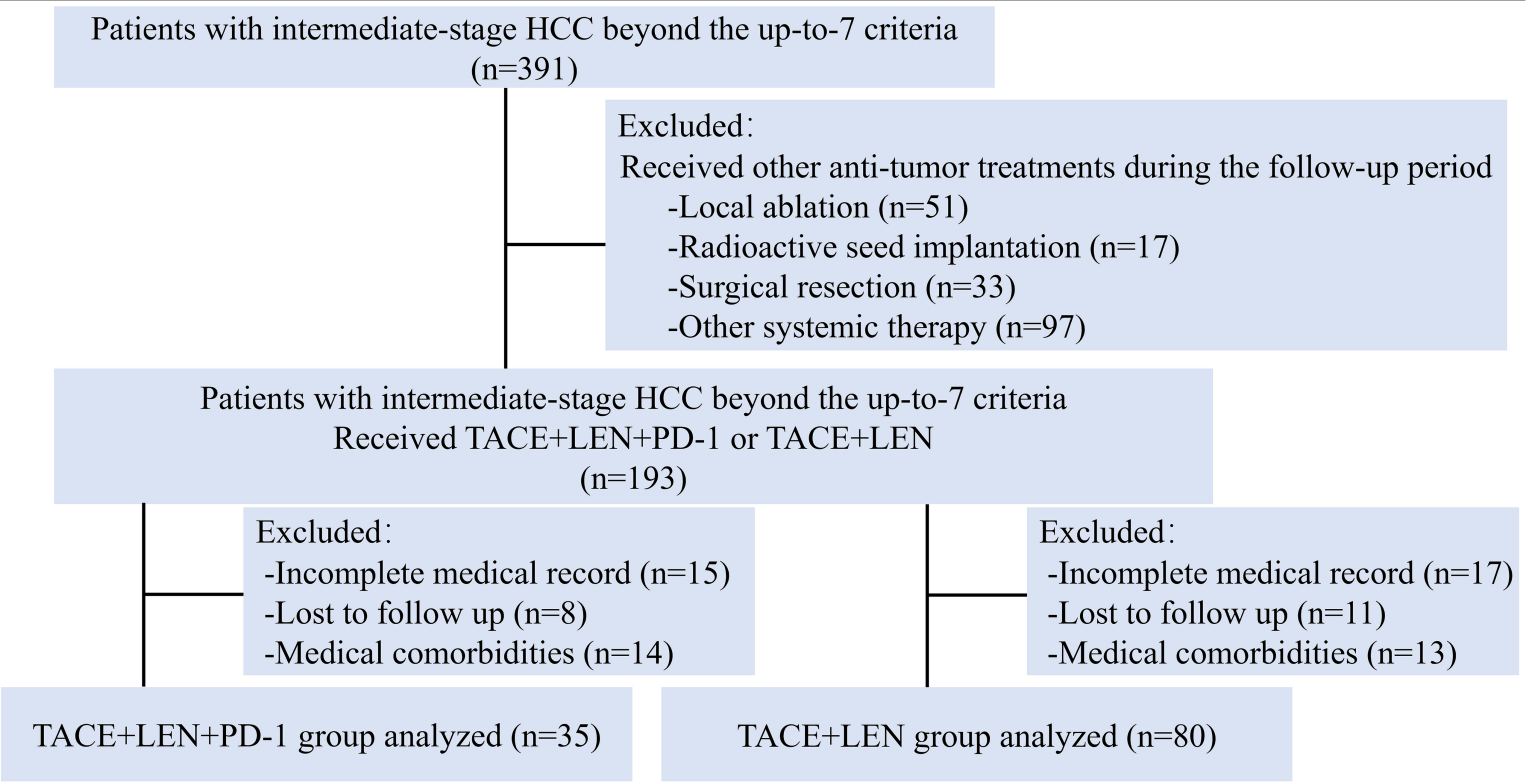
Flow diagram of patient enrollment. HCC, hepatocellular carcinoma; TACE, transarterial chemoembolization; LEN, Lenvatinib.

During the study period, a total of 391 HCC patients with intermediate-stage HCC beyond the up-to-7 criteria were screened for eligibility. Of these, 193 patients were treated with either TACE+LEN+PD-1 or TACE+LEN. A total of 78 patients were excluded based on the exclusion criteria (**Figure 1**). Finally, 115 patients were included in the analysis, with 35 in the TACE+LEN+PD-1 group and 80 in the TACE+LEN group. The baseline characteristics of the two groups were well-matched, as shown in **Table 1**.

**Table 1:** Baseline characteristics of the patients.

Characteristic	TACE+LEN+PD-1 (n =35)	TACE+LEN (n = 80)	P value
Age, years	55.7 ± 11.7	54.5 ± 13.2	0.389
Sex			0.698
Male	32	75	
Female	3	5	
BMI, kg/m2	22.2 ± 3.3	22.4 ± 3.4	0.859
ECOG PS			0.725
0	22	53	
1	13	27	
HBsAg			0.603
Positive	31	75	
Negative	4	5	
Tumor size (cm)			0.437
<10	21	54	
≥10	14	26	
Number of lesions			0.612
<4	6	17	
≥4	29	63	
TBS			0.376
Low	15	46	
High	20	34	
Child-Pugh score			0.669
5	20	46	
6	10	18	
7	5	16	
AFP, μg/L			0.509
<400	23	58	
≥400	12	22	
Sessions of TACE (range)	2.9 (2–6)	2.7 (1-5)	0.130

Data were presented as n (%) or mean ± standard deviation.

TACE, transarterial chemoembolization; LEN, lenvatinib; BMI, body mass index; ECOG PS, Eastern Cooperative Oncology Group Performance Status; HBsAg, hepatitis B surface antigen; TBS, tumor burden score; AFP, a-fetoprotein.

The mean age was 55.7 ± 11.7 years in the TACE+LEN+PD-1 group and 54.5 ± 13.2 years in the TACE+LEN group. In both groups, nearly 90% of HCC cases were associated with HBV infection. Additionally, in the TACE+LEN+PD-1 group, 15 patients (42.8%) had a low TBS while 20 patients (57.2%) had a high TBS. In the TACE+LEN group, 46 patients (57.5%) had a low TBS and 34 patients (42.5%) had a high TBS (**Table 1**). Patients in the TACE+LEN group underwent an average of 2.7 (range, 1-5) TACE sessions per patient, while those in the TACE+LEN+PD-1 group had an average of 2.9 (range, 2-6) procedures (*P*=0.130). The mean duration of lenvatinib treatment was 7.8 ± 6.7 months (range, 1.3-37.2) in the TACE+LEN+PD-1 group and 9.3 ± 5.4 months (range, 1.9-31.9) in the TACE+LEN group. The distribution of PD-1 inhibitors used in the TACE+LEN+PD-1 group was as follows: 10 patients (28.6%) received pembrolizumab, 14(40.0%) received sintilimab, 4(11.4%) received tislelizumab, and 7(20.0%) received camrelizumab.

### Survival and tumor responses

**Figure 2:**
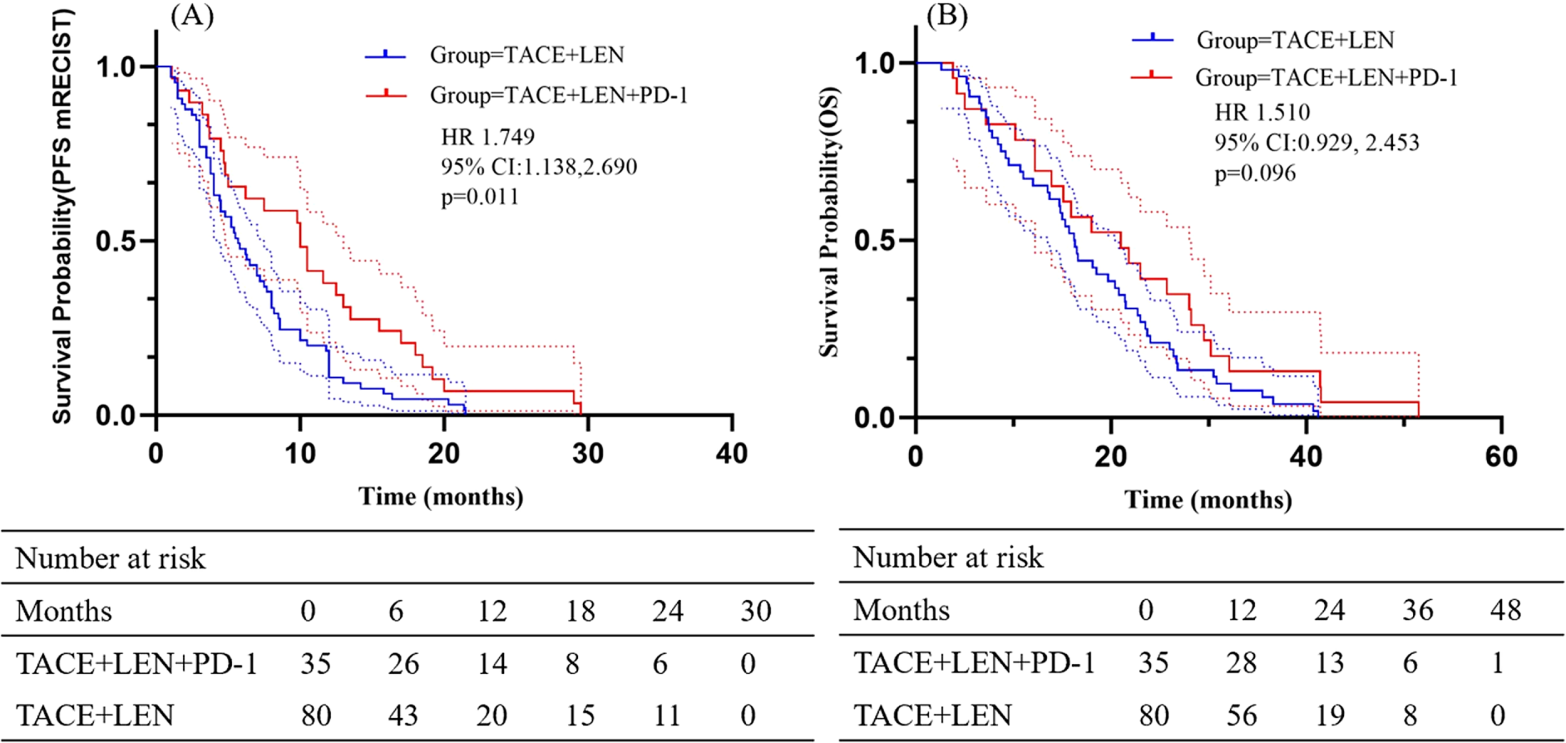
Kaplan-Meier curves of **(A)** PFS based on mRECIST and **(B)** OS for patients in the TACE+LEN+PD-1 and TACE+LEN groups. Dotted areas: 95% Cls. TACE, transarterial chemoembolization; LEN, lenvatinib; PFS, progression-free survival; OS, overall survival; RECIST, Response Evaluation.

**Figure 3:**
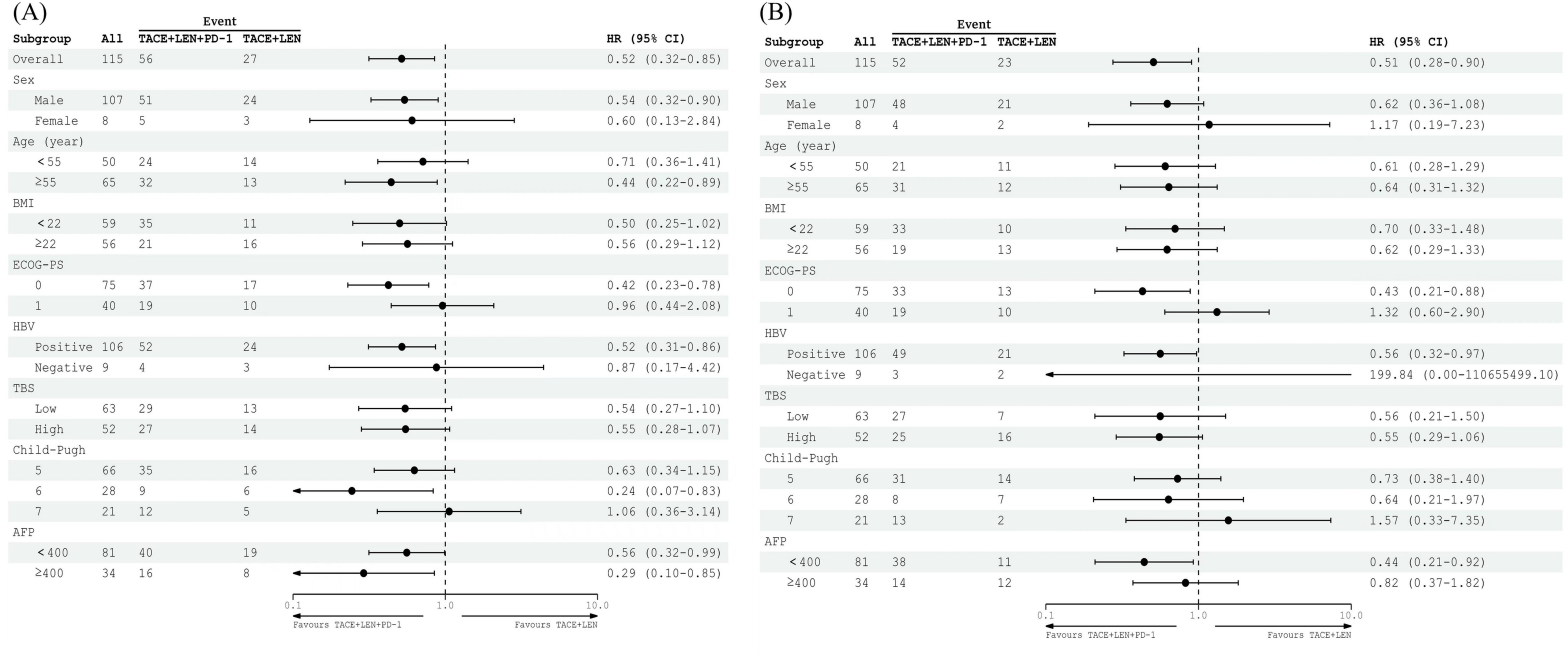
Kaplan-Meier curves of **(A)** PFS based on mRECIST and **(B)** OS for patients in the TACE+LEN+PD-1 and TACE+LEN groups. Dotted areas: 95% Cls. TACE, transarterial chemoembolization; LEN, lenvatinib; PFS, progression-free survival; OS, overall survival; RECIST, Response Evaluation.

The follow-up period ranged from 2.6 to 51.5 months, with a median of 18.9 months. During this period, 23 patients (65.7%) in the TACE+LEN+PD-1 group and 52 patients (65.0%) in the TACE+LEN group died. Kaplan-Meier survival analysis demonstrated a median PFS of 5.7 months in the TACE+LEN group compared to 10.0 months in the TACE+LEN+PD-1 group, indicating a significant improvement of 4.3 months (hazard ratio [HR]=1.749, 95% confidence interval [CI] 1.138-2.690, *P*=0.011) (**Figure 2**). The median OS was 16.2 months in the TACE+LEN group and 21.0 months in the TACE+LEN+PD-1 group, representing a numerical extension of 4.8 months, though this difference did not reach statistical significance (HR=1.510, 95% CI 0.929-2.453, *P*=0.096) (**Figure 2**). Among patients with intrahepatic tumor progression, the median PFS1 was 5.3 months in the TACE+LEN group and 10.0 months in the TACE+LEN+PD-1 group, with no statistically significant difference between the two groups (HR=1.759, 95% CI 0.949-3.259, *P*=0.072) (**Figure 3**). In patients who developed MVI or EHS, the median PFS2 was significantly longer in the TACE+LEN+PD-1 group compared to the TACE+LEN group (12.0 vs. 7.5 months; HR=1.979; 95% CI 1.127-3.476, *P*=0.007) (**Figure 3**).

Tumor responses following the first TACE procedure for both groups are summarized in **Table 2**. According to mRECIST criteria, the TACE+LEN+PD-1 group exhibited a higher ORR (82.9% vs. 73.7%, *P*=0.289) and DCR (88.6% vs. 82.5%, *P*=0.410) compared to the TACE+LEN group. However, these differences in ORR and DCR between the two groups were not statistically significant.

**Table 2:** Tumor response rates according to mRECIST.

Response, n (%)	TACE+LEN+PD-1 (n=35)	TACE+LEN (n=80)	P value
CR	1(2.8)	1(1.3)	
PR	28(80.0)	58(72.5)	
SD	2(5.7)	7(8.8)	
PD	4(11.4)	14(17.5)	
ORR	29(82.9)	59(73.7)	0.289
DCR	31(88.6)	66(82.5)	0.410

Data were presented as n (%).

TACE, transarterial chemoembolization; LEN, lenvatinib; CR, complete response; PR, partial response; SD, stable disease; PD, progressive disease; ORR, objective response rate; DCR, disease control rate.

### Prognostic factors analysis

Based on the results of the univariate and multivariate analyses (**Table 3**), treatment option (TACE+LEN vs. TACE+LEN+PD-1; hazard ratio [HR]=0.544, 95% CI 0.305-0.908, *P*=0.020) and TBS (low vs. high; HR=1.772, 95% CI 1.183-3.520, *P*=0.010) were identified as the independent prognostic factors for PFS. In addition, treatment option (TACE+LEN vs. TACE+LEN+PD-1; HR=0.519, 95% CI 0.285-0.944, *P*=0.032) and TBS (HR=1.904, 95% CI 1.092-3.320, *P*=0.023) were also identified as the independent prognostic factors for OS.

**Table 3:** Analyses of prognostic factors for survival.

Factor	Progression-free survival				Overall survival			
	Univariate analysis		Multivariate analysis		Univariate analysis		Multivariate analysis	
	HR (95% CI) P value		HR (95% CI) P value		HR (95% CI) P value		HR (95% CI) P value	
Sex								
Male/Female	0.809(0.388,1.685)	0.571			0.873(0.378,2.020)	0.752		
Age(year)								
<55/≥55	0.787(0.505,1.227)	0.291			0.872(0.549,1.383)	0.559		
BMI								
<22/≥22	0.732(0.471,1.138)	0.166			0.994(0.622,1.586)	0.979		
ECOG PS								
0/1	1.216(0.768,1.925)	0.404			1.551(0.955,2.522)	0.076		
HBsAg								
Positive/Negative	0.899(0.412,1.960)	0.789			1.399(0.561,3.489)	0.472		
TBS								
Low/High	1.480(0.945,2.319)	0.087	1.772(1.074,2.924)	0.025	1.397(0.879,2.220)	0.157	1.904(1.092,3.320)	0.023
Child-Pugh								
A/B	0.921(0.538,1.578)	0.921			1.744(0.971,3.133)	0.063		
AFP								
<400/≥400	1.695(1.037,2.773)	0.035			1.335(0.823,2.165)	0.242		
Treatment option								
TACE+L/TACE+L+P	0.545(0.338,0.878)	0.013	0.544(0.325,0.908)	0.020	0.644(0.382,1.086)	0.099	0.519(0.285,0.944)	0.032

Analyses were performed using Cox proportional hazard regression model.

HR, hazard ratio; CI, confidence interval; BMI, body mass index; ECOG PS, Eastern Cooperative Oncology Group Performance Status; HBsAg, hepatitis B surface antigen; AFP, a-fetoprotein; TBS, tumor burden score; TACE+L+P, transarterial chemoembolization combined with lenvatinib plus PD-1 inhibitor; TACE+L, transarterial chemoembolization combined with lenvatinib.

**Figure 4:**
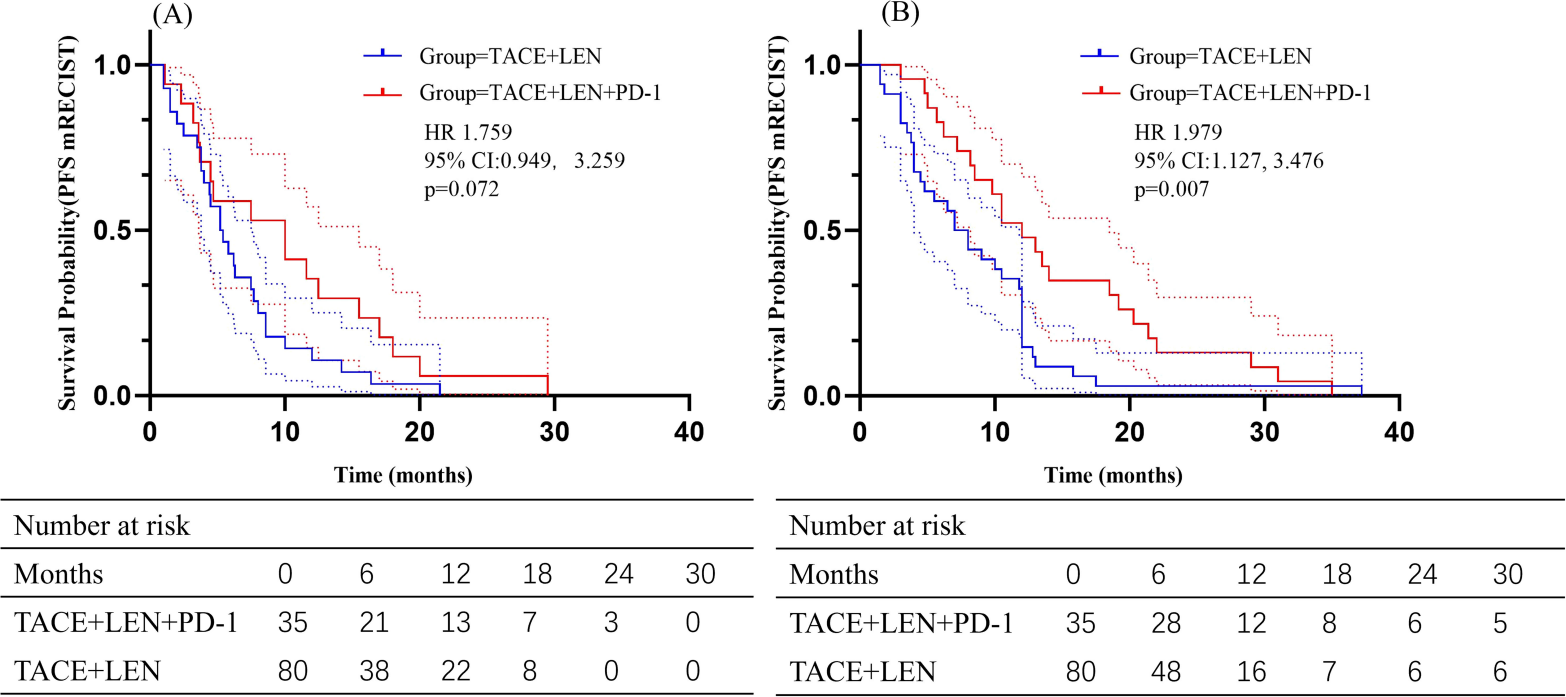
Forest plot of **(A)** PFS based on mRECIST and **(B)** OS for patients in different subgroups between the TACE+LEN+PD-1 and TACE+LEN groups. TACE, transarterial chemoembolization; LEN, lenvatinib; BMI, body mass index; ECOG PS, Eastern Cooperative Oncology Group Performance Status; HBsAg, hepatitis B surface antigen; TBS, tumor burden score; AFP, a-fetoprotein.

Subgroup analyses examining factors influencing PFS and OS revealed that the TACE+LEN+PD-1 treatment regimen offered a significant survival advantage for patients with a PS of 0 or those who were positive for HBV. However, it did not demonstrate clinical benefits in patients with a high TBS, an alpha-fetoprotein (AFP) level of ≥400 ng/mL, or a Child-Pugh score of 7. The results of the subgroup analyses for OS and PFS (mRECIST), based on baseline characteristics, are illustrated in the forest plots shown in **Figures 4A, B**.

### Safety

A total of 106 out of 115 patients (92.2%) experienced treatment-related adverse events (AEs), with no grade 5 AEs reported (**Table 4**). The frequency and severity of AEs associated with TACE were comparable between the TACE+LEN+PD-1 group and the TACE+LEN group (any grade: 88.6% vs. 91.3%, *P*=0.733; grade 3 or 4: 48.6% vs. 42.5%, *P*=0.546). Common TACE-related AEs included abdominal pain and fever, occurring in 12 (34.3%) and 10 (28.6%) patients in the TACE+LEN+PD-1 group, and in 21 (26.3%) and 17 (21.3%) patients in the TACE+LEN group, respectively (*P*=0.381 and 0.394).

**Table 4:** Treatment-related adverse events.

Adverse events	Any grade (n%)			Grade 3 or 4 (n%)		
	TACE+LEN+ PD-1 (N=35)	TACE+LEN (N=80)	P value	TACE+LEN+ PD-1 (N=35)	TACE+LEN (N=80)	P value
Related to TACE	31(89)	73(91)	0.733	17(49)	34(43)	0.546
Abdominal pain	28(80)	62(78)	0.765	12(34)	21(26)	0.381
Fever	25(71)	54(68)	0.676	10(29)	17(21)	0.394
New ascites	3(9)	6(8)	1	0	0	-
Pleural effusion	1(3)	3(4)	1	0	0	-
Inguinal hematoma	2(6)	5(6)	1	0	0	-
Biliary injury	2(6)	3(4)	0.639	1	0	-
Related to drug	29(83)	71(89)	0.388	18(51)	32(40)	0.255
Hand-foot syndrome	6(17)	15(19)	0.837	2(6)	6(8)	1
Hypertension	13(37)	30(38)	0.971	4(11)	12(15)	0.773
Nausea/Vomiting	24(69)	58(73)	0.668	9(26)	19(24)	0.821
Diarrhea	11(31)	24(30)	0.878	3(9)	8(10)	1
Decreased appetite	13(37)	22(28)	0.301	2(6)	6(8)	1
Fatigue	10(29)	21(26)	0.796	1(3)	4(5)	1
Weight Loss	9(26)	18(23)	0.828	2(6)	3(4)	0.639
Ventosity	8(23)	15(19)	0.612	0	2(3)	-
Proteinuria	7(20)	17(21)	0.879	3(9)	7(9)	1
Elevated GGT	4(11)	10(13)	1	0	2(3)	-
Elevated AST	8(23)	16(20)	0.729	0	1(1)	-
Elevated ALT	7(20)	15(19)	0.875	1(3)	2(3)	1
Elevated ALP	6(17)	13(16)	0.906	1(3)	1(1)	1
Elevated TBIL	5(14)	12(15)	0.921	1(3)	2(3)	-
Pruritus	4(11)	10(13)	1	0	1(1)	-
Rash	10(29)	8(10)	0.012	2(6)	1(1)	0.219
Neutropenia	8(23)	15(19)	0.612	1(3)	2(3)	1
Thrombocytopenia	9(26)	13(16)	0.235	1(3)	1(1)	1
Lymphopenia	5(14)	10(13)	0.936	2(6)	2(3)	0.599
Anemia	6(17)	12(15)	0.771	0	1(1)	-
Arthralgia	6(17)	8(10)	0.417	0	0	-
Gingival bleeding	5(14)	11(14)	0.939	0	0	1
Dysphonia	4(11)	9(11)	1	0	1(1)	-
Elevated uric acid	3(9)	7(9)	1	0	0	-
Infusion reaction	3(9)	6(8)	1	1(3)	1	1
Hyperglycemia	2(6)	5(6)	1	1(3)	1(1)	1
Pneumonitis	6(17)	3(4)	0.022	2(6)	0	-
Hypothyroidism	5(14)	2(3)	0.083	1(3)	0	-
Hyperthyroidism	3(9)	1(1)	0.027	0	0	-

TACE, transarterial chemoembolization; LEN, lenvatinib.

Similarly, the incidence and severity of drug-related AEs were akin between the two groups (any grade: 82.9% vs. 88.8%, *P*=0.388; grade 3 or 4: 51.4% vs. 40.0%, *P*=0.255). In the TACE+LEN+PD-1 group, the rates of any grade AEs and grade 3–4 AEs associated with lenvatinib were comparable to those in the TACE+LEN group. However, the occurrence of any grade immune-related adverse events (irAEs), such as pneumonitis and hyperthyroidism, was significantly higher in the TACE+LEN+PD-1 group compared to the TACE+LEN group (*P*=0.022 and 0.027, respectively). There were no significant differences noted in grade 3 or 4 AEs. Notably, no patients in either group experienced treatment-related mortality.

## Discussion

In this study, we observed that TACE+LEN+PD-1 provided a significant PFS advantage over TACE+LEN in patients with intermediate-stage HCC exceeding the up-to-7 criteria. This advantage was evidenced by an increase in median PFS from 5.7 to 10.0 months, particularly in patients who developed MVI or EHS. Furthermore, the TACE+LEN+PD-1 group showed improved OS (from 16.2 to 21.0 months), ORR, and DCR compared to the TACE+LEN group, though these differences did not reach statistical significance. Univariate and multivariate analyses identified the addition of a PD-1 inhibitor and TBS as independent predictors of OS and PFS between the two groups. Subgroup analyses revealed that the addition of a PD-1 inhibitor was particularly beneficial in patients with an ECOG PS of 0 or those who were HBV positive. However, the addition of a PD-1 inhibitor did not show benefits in patients with a Child-Pugh score of 7. Importantly, the addition of a PD-1 inhibitor did not introduce additional safety concerns in the TACE+LEN+PD-1 group. In conclusion, the combination of TACE, lenvatinib, and a PD-1 inhibitor may offer synergistic antitumor activity, contributing to improved clinical outcomes in patients with intermediate-stage HCC exceeding the up-to-7 criteria.

A key finding of our study was the significant prolongation of time from the first TACE session to stage progression, demonstrating that the addition of PD-1 inhibitors not only enhances PFS but also delays the development of more aggressive disease features. This effect is likely attributable to the ability of PD-1 inhibitors to activate T cells, thereby enhancing the immune system’s capacity to eliminate tumor cells (19, 20). Immune activation induced by PD-1 inhibitors can act as a ‘supplementary strike’ against residual tumor cells following TACE+LEN treatment. This may effectively delay the onset of aggressive disease characteristics, such as MVI or EHS (21, 22). The addition of PD-1 inhibitors significantly extended the time to stage progression. This allowed patients to remain in the intermediate stage of HCC for a longer period, preserving liver function and providing opportunities for repeated TACE or systemic therapies.

Recently, several studies have explored the efficacy and safety of TACE+LEN+PD-1 versus TACE+LEN across different HCC subtypes. In patients with intermediate-stage HCC beyond the up-to-11 criteria, one study demonstrated that the TACE+LEN+PD-1 group achieved significantly longer PFS (8.5 months) and OS (31.5 months) compared to the TACE+LEN group (PFS: 5.5 months, OS: 20.5 months) (23). Similarly, another study found that in patients with unresectable recurrent HCC meeting the up-to-7 criteria, the median PFS was markedly longer in the TACE+LEN+PD-1 group (24.1 months) compared to the TACE+LEN group (17.3 months, *P*< 0.001) (24). Furthermore, in advanced HCC, another study reported that the TACE+LEN+PD-1 group achieved a PFS of 7.3 months and an OS of 16.9 months, outperforming the TACE+LEN group (PFS: 4.0 months, OS: 12.1 months) (13). However, the difference in PFS and OS compared with previous research may be explained by higher tumor burden and different CNLC stage in our study. In our cohort, over 50% of patients had a high TBS, a validated metric for predicting survival outcomes in intermediate-stage patients undergoing TACE (17). Elevated TBS is often associated with more aggressive tumor biology, which may adversely impact treatment efficacy and overall outcomes (25).

As was shown in our study, the addition of PD-1 inhibitors and TBS was identified as an independent risk factor for PFS and OS in intermediate HCC patients exceeding the up-to-7 criteria. More importantly, subgroup analyses revealed that TACE+LEN+PD-1 treatment led to a prolonged OS in patients with a PS of 0 or those who were positive for HBV. The potential reasons are as follows: First, for patients with a PS of 0, their better baseline status allows for higher tolerance to the TACE+LEN+PD-1 therapy, leading to improved outcomes (26). Second, in our study, nearly 90% of patients have an HBV background and severe liver cirrhosis, which increases the risk of liver failure after HCC treatment (27, 28). This finding may reflect the immune modulation induced by the PD-1 inhibitor, which could enhance the antitumor response in the context of HBV. The immunological environment in HBV-related HCC may make it more responsive to PD-1 inhibitors, leading to improved outcomes when combined with TACE and lenvatinib (29, 30). However, the lack of benefit observed in patients with a Child-Pugh score of 7 is notable. This finding underscores the importance of assessing liver function in the treatment decision-making process, as patients with more advanced liver disease may not be optimal candidates for the triple-combination therapy in intermediate HCC patients exceeding the up-to-7 criteria.

Based on the results of our study, although irAEs were more frequent in the TACE+LEN+PD-1 group, all AEs were manageable and consistent with previously reported data for each individual treatment (12, 17, 23, 31). No new or unexpected AEs were observed. Furthermore, the incidence and severity of AEs in the TACE+LEN+PD-1 group were comparable to those in the TACE+LEN group. These findings suggest that both TACE+LEN+PD-1 and TACE+LEN treatments are well-tolerated, and the addition of a PD-1 inhibitor to TACE+LEN does not significantly increase the risk of AEs compared to TACE+LEN, demonstrating an acceptable safety profile for TACE+LEN+PD-1. These results further support the feasibility and safety of TACE+LEN+PD-1 for the treatment of intermediate HCC patients exceeding the up-to-7 criteria.

Despite the favorable therapeutic responses and survival outcomes observed in this study, there are several limitations that need to be addressed in future research. Firstly, although this single-center study and the involvement of a unified interventional surgery team may reduce selection bias, the retrospective nature of this study may still introduce inherent biases, as treatment decisions were based on the individual preferences of both the attending physician and the patient. This may affect the generalizability of the findings. Secondly, for the treatment of intermediate HCC exceeding the up-to-7 criteria, the triple combination therapy is not yet a standard recommendation and requires further investigation in larger, randomized controlled trials to confirm its safety and efficacy. Additionally, the relatively small sample size in this study limits the statistical power, and the follow-up period was insufficient to assess long-term outcomes such as PFS and OS. As a result, the results of subgroup analyses should be interpreted with caution. Lastly, larger-scale, prospective studies with extended follow-up periods are needed to validate our findings and provide stronger evidence for clinical decision-making.

In conclusion, our study showed safety and promising outcomes with the treatment of TACE+LEN+PD-1 in intermediate HCC patients exceeding the up-to-7 criteria. While the triple combination therapy has the potential to redefine treatment paradigm, the optimal patient population for its application still requires further investigation.

## Data Availability

The raw data supporting the conclusions of this article will be made available by the authors, without undue reservation.

## References

[1] Qin Y Tang C Li J Gong J. Liver cancer in China: the analysis of mortality and burden of disease trends from 2008 to 2021. BMC Cancer. (2024) 24:594. doi: 10.1186/s12885-024-12334-2 38750424 PMC11097423

[2] Xie DY Ren ZG Zhou J Fan J Gao Q. Chinese clinical guidelines for the management of hepatocellular carcinoma: updates and insights. Hepatobiliary Surg Nutr. (2019) 9:452–63. doi: 10.21037/hbsn-20-480 PMC7423548 32832496

[3] Hung YW Lee IC Chi CT Lee RC Liu CA Chiu NC. Redefining tumor burden in patients with intermediate-stage hepatocellular carcinoma: the seven-eleven criteria. Liver Cancer. (2021) 10:629–40. doi: 10.1159/000517393 PMC8647089 34950185

[4] Mazzaferro V Llovet JM Miceli R Bhoori S Schiavo M Mariani L. Predicting survival after liver transplantation in patients with hepatocellular carcinoma beyond the Milan criteria: a retrospective, exploratory analysis. Lancet Oncol. (2009) 10:35–43. doi: 10.1016/S1470-2045(08)70284-5 19058754

[5] Koroki K Ogasawara S Ooka Y Kanzaki H Kanayama K Maruta S. Analyses of intermediate-stage hepatocellular carcinoma patients receiving transarterial chemoembolization prior to designing clinical trials. Liver Cancer. (2020) 9:596–612. 33083283 10.1159/000508809 PMC7548915

[6] Kudo M Han KH Ye SL Zhou J Huang YH Lin SM. A changing paradigm for the treatment of intermediate-stage hepatocellular carcinoma: asia-pacific primary liver cancer expert consensus statements. Liver Cancer. (2020) 9:245–60. doi: 10.1159/000507370 PMC7325125 32647629

[7] Kudo M Ueshima K Ikeda M Torimura T Tanabe N Aikata H. Final results of TACTICS: A randomized, prospective trial comparing transarterial chemoembolization plus sorafenib to transarterial chemoembolization alone in patients with unresecta ble hepatocellular carcinoma. Liver Cancer. (2022) 11:354–67. doi: 10.1159/000522547 PMC9294961 35978604

[8] Kudo M Finn RS Qin S Han KH Ikeda K Piscaglia F. Lenvatinib versus sorafenib in first-line treatment of patients with unresecta ble hepatocellular carcinoma: a randomised phase 3 non-inferiority trial. Lancet. (2018) 391:1163–73. 10.1016/S0140-6736(18)30207-1 29433850

[9] Peng Z Fan W Zhu B Wang G Sun J Xiao C. Lenvatinib combined with transarterial chemoembolization as first-line treatment for advanced hepatocellular carcinoma: A phase III, randomized clinical trial (LAUNCH). J Clin Oncol. (2023) 41:117–27. doi: 10.1200/JCO.22.00392 35921605

[10] Kudo M Ueshima K Saeki I Ishikawa T Inaba Y Morimoto N. A phase 2, prospective, multicenter, single-arm trial of transarterial chemoembolization therapy in combination strategy with lenvatinib in patients with unresecta ble intermediate-stage hepatocellular carcinoma: TACTICS-L trial. Liver Cancer. (2023) 13:99–112. 38344448 10.1159/000531377 PMC10857829

[11] Finn RS Qin S Ikeda M Galle PR Ducreux M Kim TY. Atezolizumab plus Bevacizumab in Unresect able Hepatocellular Carcinoma. N Engl J Med. (2020) 382:1894–905. doi: 10.1056/NEJMoa1915745 32402160

[12] Finn RS Ikeda M Zhu AX Sung MW Baron AD Kudo M. Phase ib study of lenvatinib plus pembrolizumab in patients with unresect able hepatocellular carcinoma. J Clin Oncol. (2020) 38:2960–70. doi: 10.1200/JCO.20.00808 PMC7479760 32716739

[13] Cai M Huang W Huang J Shi W Guo Y Liang L. Transarterial chemoembolization combined with lenvatinib plus PD-1 inhibitor for advanced hepatocellular carcinoma: A retrospective cohort study. Front Immunol. (2022) 13:848387. doi: 10.3389/fimmu.2022.848387 35300325 PMC8921060

[14] Kumar D Neeman E Zhu S Sun H Kotak D Liu R. Revisiting the association of ECOG performance status with clinical outcomes in diverse patients with cancer. J Natl Compr Canc Netw. (2024) 22:e237111. doi: 10.6004/jnccn.2023.7111 38653321

[15] Palmieri C Macpherson I. Use of the Child-Pugh score in anticancer drug dosing decision making: proceed with caution. Lancet Oncol. (2019) 20:e289. doi: 10.1016/S1470-2045(19)30296-7 31162096

[16] Clinical Guidelines Committee of Chinese College of Interventionalists. Chinese clinical practice guidelines for transarterial chemoembolization of hepatocellular carcinoma (2023 edition). Zhonghua Yi Xue Za Zhi. (2023) 103:2674–94. 10.3760/cma.j.cn112137-20230630-01114 37675541

[17] Ho SY Liu PH Hsu CY Ko CC Huang YH Su CW. Tumor burden score as a new prognostic marker for patients with hepatocellular carcinoma undergoing transarterial chemoembolization. J Gastroenterol Hepatol. (2021) 36:3196–203. doi: 10.1111/jgh.v36.11 34159651

[18] Llovet JM Lencioni R. mRECIST for HCC: Performance and novel refinements. J Hepatol. (2020) 72:288–306. doi: 10.1016/j.jhep.2019.09.026 31954493 PMC12452114

[19] Im SJ Hashimoto M Gerner MY Lee J Kissick HT Burger MC. Defining CD8+ T cells that provide the proliferative burst after PD-1 therapy. Nature. (2016). doi: 10.1038/nature19330 PMC5297183 27501248

[20] Magen A Hamon P Fiaschi N Soong BY Park MD Mattiuz R. Intratumoral dendritic cell-CD4^+^ T helper cell niches enable CD8^+^ T cell differentiation following PD-1 blockade in hepatocellular carcinoma. Nat Med. (2023) 29:1389–99. doi: 10.1038/s41591-023-02345-0 PMC11027932 37322116

[21] Chakraborty E Sarkar D. Emerging therapies for hepatocellular carcinoma (HCC). Cancers (Basel). (2022) 14:2798. 35681776 10.3390/cancers14112798 PMC9179883

[22] Sun L Xu X Meng F Liu Q Wang H Li X. Lenvatinib plus transarterial chemoembolization with or without immune checkpoint inhibitors for unresect able hepatocellular carcinoma: A review. Front Oncol. (2022) ;12:980214. 36249023 10.3389/fonc.2022.980214 PMC9555078

[23] Chen S Shuangyan T Shi F Cai H Wu Z Wang L. TACE plus lenvatinib and tislelizumab for intermediate-stage hepatocellular carcinoma beyond up-to-11 criteria: a multicenter cohort study. 10.3389/fimmu.2024.1430571 PMC11310062 39131156

[24] Wang WJ Liu ZH Wang K Yu HM Cheng YQ Xiang YJ. Efficacy and safety of TACE combined with lenvatinib and PD-1 inhibitors for unresecta ble recurrent HCC: A multicenter, retrospective study. Cancer Med. (2023) 12:11513–24. doi: 10.1002/cam4.v12.10 PMC10242311 36999793

[25] Kudo M. A new treatment option for intermediate-stage hepatocellular carcinoma with high tumor burden: initial lenvatinib therapy with subsequent selective TACE. Liver Cancer. (2019) 8:299–311. doi: 10.1159/000502905 31768341 PMC6872999

[26] Han CL Tian BW Yan LJ Ding ZN Liu H Pan GQ. The effect of age, sex, and eastern cooperative oncology group performance status on the efficacy and safety of immune checkpoint inhibitors in patients with hepatocellular carcinoma: a systematic review and meta-analysis. Expert Rev Anticancer Ther. (2024) 24:303–12. doi: 10.1080/14737140.2024.2341723 38623811

[27] Liu J Yu Y Zhao H Guo L Yang W Yan Y. Latest insights into the epidemiology, characteristics, and therapeutic strategies of chronic hepatitis B patients in indeterminate phase. Eur J Med Res. (2024) 29:343. doi: 10.1186/s40001-024-01942-0 38902822 PMC11191257

[28] Zamor PJ deLemos AS Russo MW. Viral hepatitis and hepatocellular carcinoma: etiology and management. J Gastrointest Oncol. (2017) 8:229–42. doi: 10.21037/jgo.2017.03.14 PMC5401856 28480063

[29] Ding Z Fang G Tang Y Zeng Y. The impact of PD-1 inhibitors on prognosis in unresecta ble hepatocellular carcinoma treated with TACE and lenvatinib: a retrospective study. Sci Rep. (2024) 14:14334. doi: 10.1038/s41598-024-63571-1 38906915 PMC11192886

[30] Li Z Han N Ren X Zhang Y Chu X. Effectiveness of TKI inhibitors combined with PD-1 in patients with postoperative early recurrence of HCC: A real-world study. Front Oncol. (2022) 12:833884. doi: 10.3389/fonc.2022.833884 35433466 PMC9008361

[31] Xin Y Zhang X Liu N Peng G Huang X Cao X. Efficacy and safety of lenvatinib plus PD-1 inhibitor with or without transarterial chemoembolization in unresecta ble hepatocellular carcinoma. Hepatol Int. (2023) 17:753–64. doi: 10.1007/s12072-023-10502-3 37038024

